# Cardiac telocytes are double positive for CD34/PDGFR-α

**DOI:** 10.1111/jcmm.12615

**Published:** 2015-06-17

**Authors:** Qiulian Zhou, Lei Wei, Chongjun Zhong, Siyi Fu, Yihua Bei, Radu-Ionuț Huică, Fei Wang, Junjie Xiao

**Affiliations:** a Regeneration and Ageing Lab, Experimental Center of Life Sciences, School of Life Science, Shanghai UniversityShanghai, China; b Department of Thoracic and Cardiovascular Surgery, The First Affiliated Hospital of Nanjing Medical UniversityNanjing, China; c Department of Thoracic and Cardiovascular Surgery, The Second Affiliated Hospital of NanTong UniversityNantong, China; d Department of Immunopathology, Victor Babeş National Institute of PathologyBucharest, Romania; e Division of Gastroenterology and Hepatology, Digestive Disease Institute, Tongji Hospital, Tongji University School of MedicineShanghai, China

**Keywords:** telocytes, myocardium, CD34, PDGFR-α, β, interstitial cells, cardiac repair

## Abstract

Telocytes (TCs) are a distinct type of interstitial cells, which are featured with a small cellular body and long and thin elongations called telopodes (Tps). TCs have been widely identified in lots of tissues and organs including heart. Double staining for CD34/PDGFR-β (Platelet-derived growth factor receptor β) or CD34/Vimentin is considered to be critical for TC phenotyping. It has recently been proposed that CD34/PDGFR-α (Platelet-derived growth factor receptor α) is actually a specific marker for TCs including cardiac TCs although the direct evidence is still lacking. Here, we showed that cardiac TCs were double positive for CD34/PDGFR-α in primary culture. CD34/PDGFR-α positive cells (putative cardiac TCs) also existed in mice ventricle and human cardiac valves including mitral valve, tricuspid valve and aortic valve. Over 87% of cells in a TC-enriched culture of rat cardiac interstitial cells were positive for PDGFR-α, while CD34/PDGFR-α double positive cells accounted for 30.25% of the whole cell population. We show that cardiac TCs are double positive for CD34/PDGFR-α. Better understanding of the immunocytochemical phenotypes of cardiac TCs might help using cardiac TCs as a novel source in cardiac repair.

## Introduction

Telocytes (TCs), a distinct type of interstitial cells (for more details see www.telocytes.com), have a small cellular body and long and thin elongations called telopodes (Tps) 1. Telocytes have been widely identified to be distributed in lots of tissues and organs including heart, liver, aorta, urinary bladder, bone marrow, vasculature, lung, uterine, oviduct, placenta, gastrointestinal tract *etc*. b–15. With heart, TCs have been reported in atrium, ventricle, endocardium, myocardium, epicardium, and heart valves 16–23. Their detailed roles in heart have been well summarized in a recent review 24. Telocytes are different from other types of interstitial cells as evidenced by ultrastructural characteristics, immunohistochemical features, gene profiles, proteome features and miRNA signatures 1,8,25–32. Cardiac TCs are the best characterized one at present 25.

To identify TCs, transmission electron microscopy (TEM) is generally considered to be a golden standard method 33. Although not a single immunostaining marker for TCs is specific, double immunolabelling for TCs is essentially important to distinguish them from other types of interstitial cells 34–36. Moreover, double immunolabelling is also a useful tool for semi-quantitative data analysis 25. Cardiac TCs have been reported to be positive for CD34/c-Kit, CD34/Vimentin and CD34/PDGFR-β 25. Double positive immunostaining for CD34/PDGFR-α has been identified throughout different segments of human gut, including large and small intestine, corpus and antrum, gastric fundus, and oesophagus 7,37. In addition, in mice and human liver, TCs are also found to be positive for CD34/PDGFR-α b,^38^–^40^. Interestingly, in the human bladder and uterine, CD34/PDGFR-α positive TCs also exist 4,41. Recently, CD34/PDGFR-α double positive immunostaining has been suggested to be a marker for cardiac TCs, however, the direct evidence is still lacking 25.

In this study, cardiac TCs were isolated from adult mice and confirmed by double positive immunostaining for CD34/Vimentin and CD34/PDGFR-β. Based on double labelling for CD34 and PDGFR-α, we confirmed that isolated cardiac TCs were double positive for CD34/PDGFR-α. In addition, CD34/PDGFR-α positive cells (putative cardiac TCs) were also identified in mice ventricle and human cardiac valves including mitral valve, tricuspid valve and aortic valve. Quantitatively, CD34/PDGFR-α positive cells accounted for a third of TC-enriched rat cardiac interstitial cell population. Collectively, the present study firstly reported that cardiac TCs were double positive for CD34/PDGFR-α.

## Materials and methods

### Animal and human samples

Adult male C57BL/6 mice purchased from the animal research centre of Fudan University were used in this study. All animal experiments were conducted under the guidelines on the use and care of laboratory animals for biomedical research published by National Institutes of Health (No. 85-23, revised 1996). This study was approved by the committee on the Ethics of Animal Experiments of Shanghai University.

Rat heart samples were obtained from 6-month-old, healthy male Wistar rats weighing 270–320 g, following anterior thoracotomy.

Human heart valves were obtained from prospective multiorgan donors who did not have cardiovascular pathology in cases in which technical reasons prevented transplantation. Studies were performed under an institutional review board-approved protocol. The investigation conforms to the principles that are outlined in the Declaration of Helsinki regarding the use of human tissues.

### Cardiac TCs isolation and culture

Cardiac TCs were isolated as our previously described 30. Briefly, mice and rat hearts were minced into 1 mm^3^ pieces and incubated on an orbital shaker at 37°C for 35 min. with 0.25 mg/ml collagenase typeII (17101-015; Invitrogen, Paisley, Renfrewshire, UK). After cultured for 2 hrs to allow cardiac fibroblasts to attach, remaining suspended cells were collected and cultured in DMEM/F12 supplemented with 10% FBS, 100 U/ml penicillin, and 100 μg/ml streptomycin. Cell cultures were examined using inverted biological microscope (BM-37XC; Shanghai BM optical instruments, LTD, Shanghai, China), and TCs were photographed under 100× magnification at 48 hrs after seeded.

### Double immunofluorescent staining for CD34/Vimentin or PDGFR-β or PDGFR-α

To confirm the successful isolation of cardiac TCs, double immunofluorescent staining for CD34/PDGFR-β or CD34/Vimentin was used as our previously reported 30. Meanwhile, CD34/PDGFR-α double labelling was performed to check if isolated cardiac TCs are double positive immunostaining for CD34/PDGFR-α. CD34/PDGFR-α double labelling was also used in mice ventricle and human cardiac valves including mitral valve, tricuspid valve and aortic valve to identify the existence of CD34/PDGFR-α positive cells (putative cardiac TCs) in heart.

Cells were washed with PBS for three times and fixed in 4% paraformaldehyde for 30 min. and then permeabilized with 0.5% Triton X-100 for 30 min. Cells were then washed with PBS and blocked in 3% bovine serum albumin (BSA) for 1 hr. For tissues, frozen sections of 6 μm thickness were fixed in 4% paraformaldehyde containing 0.05% Triton X-100 for 20 min. After three times wash with PBS, they were preincubated for 1 hr in 5% BSA. Cells or mouse sections were incubated at 4°C overnight with rat monoclonal anti-CD34 (1:100, ab8158; Abcam, Cambridge, MA, USA) and rabbit polyclonal anti-PDGFR-α (1:100, ab61219; Abcam). Cells or mouse sections were incubated then with goat anti-rat FITC-labelled (1:200, sc-2011; Santa Cruz Biotechnology, Santa Cruz, CA, USA) and donkey anti-rabbit rhodamine-labelled (1:200, sc-2095; Santa Cruz Biotechnology) secondary antibodies for 2 hrs, and then were stained with DAPI (F36924; Life Technology, Grand Island, NY, USA). Human sections were incubated at 4°C overnight with mouse monoclonal anti-CD34 (1:100, ab54208; Abcam) and rabbit polyclonal anti-PDGFR-α (1:100, ab61219; Abcam, Epitomics). Human sections were incubated with goat anti-mouse (also reactive to rat) Cy3-labelled (1:200, 115-165-166; Jackson Immunoresearch Laboratories, West Grove, PA, USA) and donkey anti-rabbit FITC-labelled (1:200, 711-545-152; Jackson Immunoresearch Laboratories) secondary antibodies for 2 hrs, and then were stained with DAPI. Similar protocols were used for double immunofluorescent staining for CD34/PDGFR-β (1:100, rabbit monoclonal anti-PDGFR-β, ab32570; Abcam) or CD34/Vimentin (1:100, rabbit monoclonal anti-Vimentin, ab92547; Abcam). All images from cells were taken under a magnification of 200× with fluorescent inverted microscope (Leica DMI4000 B; Leica, Wetzlar, Germany). All images from tissues were taken using confocal laser scanning microscope (LSM 710; Carl Zeiss MicroImaging GmbH, Jena, Germany) and double immunofluorescent staining were merged using Zen 2011 software (Carl Zeiss MicroImaging GmbH).

### Flow cytometry of TC-enriched culture of rat heart interstitial cells

Cell phenotype in a TC-enriched culture of rat heart interstitial cells was assessed by labelling with Alexa Fluor 405-conjugated anti-CD34 monoclonal antibodies, PE-conjugated anti-PDGFR-α (C20) and FITC-conjugated anti-PDGFR-β (958) polyclonal antibodies (Santa Cruz Biotechnology Inc., Heidelberg, Germany).

Analysis was performed with a FACSCanto II cytometer (BD Biosciences, San Jose, CA, USA). Unstained cells were used as control. Non-specific fluorescence signals caused by spectral overlapping were automatically compensated for using BD CompBeads particles. Data acquisition and analysis were performed with BD FACSDiva 6.1 software. Evaluation of cell expression of CD34, PDGFR-α and PDGFR-β was performed with two methods: (*i*) analysis of the fluorescence signal shift, as expressed by the mean fluorescence intensity (MFI) difference between test and control samples and (*ii*) the per cent positive method, with a 5% positive threshold in the control samples.

## Results

### Cardiac TCs are double positive immunostaining for CD34/PDGFR-α *in vitro*

**Figure 1:**
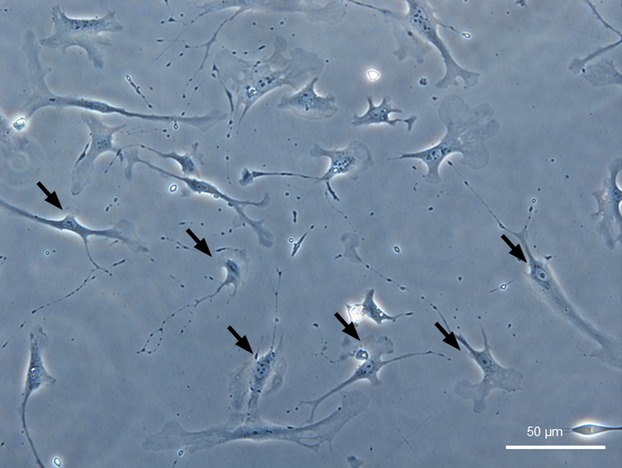
Cardiac telocytes (TCs) are showed under light microscope in primary culture. Arrows show typical TCs with long and thin telopodes with lots of dilations. Original magnification 100×; scale bar = 50 μm.

Cardiac TCs were successfully isolated as our previous reported 30. These cardiac TCs have special characteristics including relative small cell body and very long and thin Tps with lots of dilations (Fig.1). To further confirm that the cells we isolated were TCs, double immunofluorescent staining for CD34/Vimentin or CD34/PDGFR-β was used. We found that these cells were double positive for CD34/Vimentin or CD34/PDGFR-β (Fig.b), indicating that these cells were cardiac TCs.

**Figure 2:**
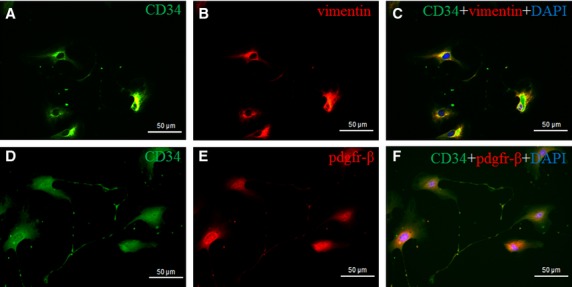
Cardiac telocytes (TCs) are CD34/Vimentin, and/or CD34/PDGFR-β double positive. Double immunofluorescence labelling for CD34 (A, green) and Vimentin (B, red) with DAPI (blue) counterstain for nuclei. TCs are CD34 and Vimentin positive (C); scale bar = 50 μm. Double immunofluorescence labelling for CD34 (D, green) and PDGFR-β (E, red) with DAPI (blue) counterstain for nuclei. TCs are CD34 and PDGFR-β positive (F); scale bar = 50 μm.

Based on the confirmation of isolation of cardiac TCs, double labelling for CD34 and PDGFR-α was performed in these cells. We found that cardiac TCs were also double positive immunostaining for CD34/PDGFR-α (Fig.c), supporting the hypothesis recently reported 25.

**Figure 3:**
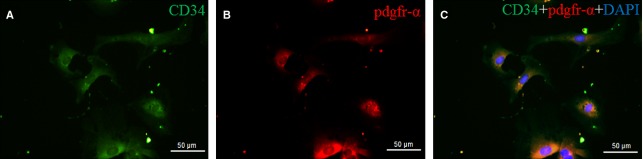
Cardiac telocytes (TCs) are CD34/PDGFR-α double positive *in vitro*. Double immunofluorescence labelling for CD34 (A, green) and PDGFR-α (B, red) with DAPI (blue) counterstain for nuclei. TCs are CD34 and PDGFR-α positive (C); scale bar = 50 μm.

### CD34/PDGFR-α positive cells (putative cardiac TCs) exist *in vivo*

**Figure 4:**
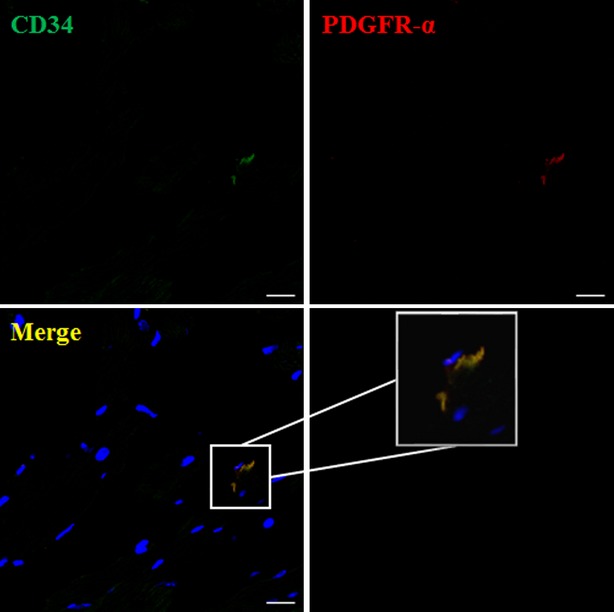
CD34/PDGFR-α positive cells (putative cardiac TCs) exist in mice ventricle. Double immunofluorescence labelling for CD34 (green) and PDGFR-α (red) with DAPI (blue) counterstain for nuclei shows that CD34/PDGFR-α positive cells (putative cardiac TCs) exist in mice ventricle; scale bar = 20 μm.

**Figure 5:**
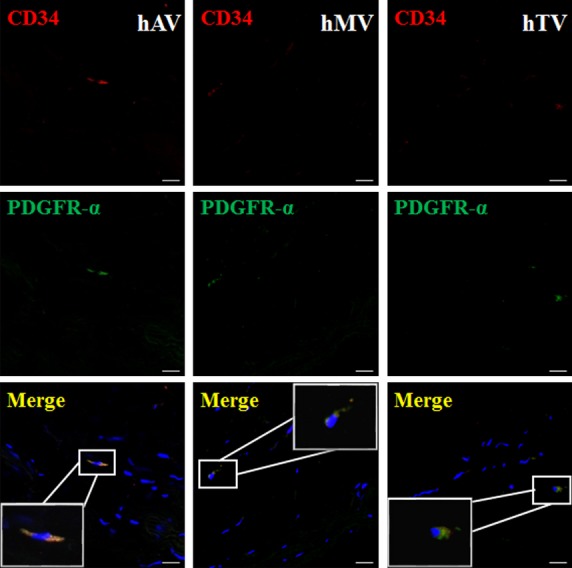
CD34/PDGFR-α positive cells (putative cardiac TCs) exist in human cardiac valves. Double immunofluorescence labelling for CD34 (red) and PDGFR-α (green) with DAPI (blue) counterstain for nuclei shows that CD34/PDGFR-α positive cells (putative cardiac TCs) exist in human atrium and cardiac valves including mitral valve (hMV), tricuspid valve (hTV) and aortic valve (hAV); scale bar = 20 μm.

To further explore if CD34/PDGFR-α positive cells (putative cardiac TCs) exist, we checked the samples from mice ventricle firstly. We identified CD34/PDGFR-α positive cells (putative cardiac TCs) in mice ventricle (Fig.4). Besides, we also explored their existence in human cardiac valves. We found that CD34/PDGFR-α positive cells (putative cardiac TCs) existed in human cardiac valves including mitral valve, tricuspid valve and aortic valve (Fig.5).

### CD34/PDGFR-α positive cells in rats by flow cytometry

**Figure 6:**
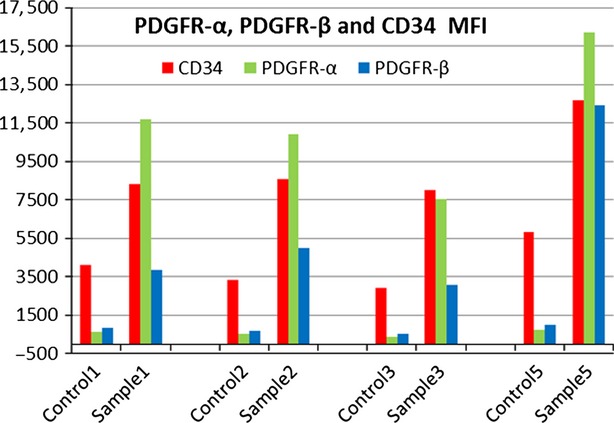
Mean fluorescence intensity (MFI) shift (expressed in relative fluorescence units) in a test *versus* control samples comparison for CD34 (red), PDGFR-α (green) and PDGFR-β (blue). MFI values for CD34, PDGFR-α, and PDGFR-β positive cells respectively are shown for four experiments with TC-enriched stromal cells from rat heart.

For flow cytometry analysis of TC-enriched rat cardiac interstitial cells, the average MFI difference between test and control samples were calculated: CD34 Alexa Fluor 405: 5348 relative fluorescence units (RFU), PDGFR-α PE: 11034 RFU, PDGFR-β FITC: 5316 RFU (Fig.6).

**Figure 7:**
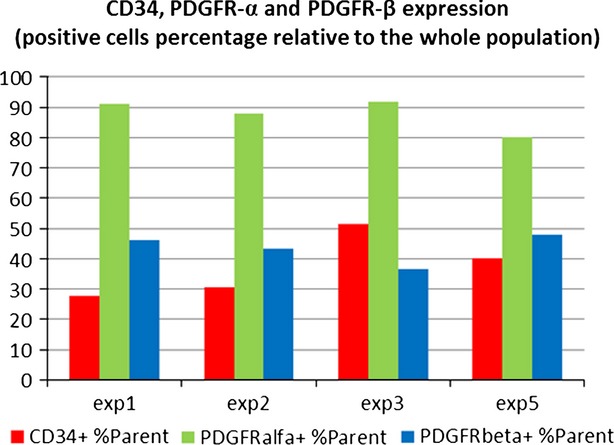
CD34, PDGFR-α and PDGFR-β expression (positive cells percentages relative to the whole population in TC-enriched stromal cells from rat heart). The relative expression of three markers is shown for four experiments, as percentage of positive cells for CD34, PDGFR-α and PDGFR-β respectively, in relation to the whole (‘Parent’) cell population.

**Figure 8:**
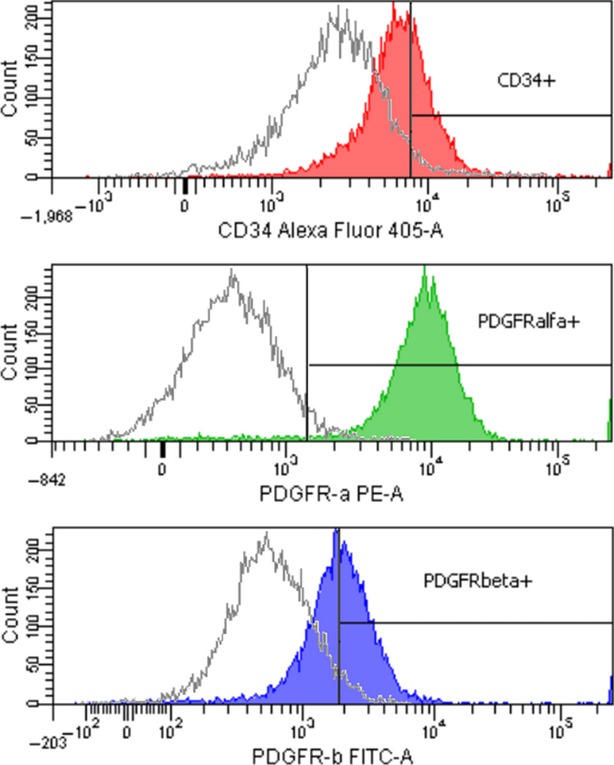
Flow cytometry histograms showing CD34, PDGFR-α and PDGFR-β expression for TC-enriched stromal cells from rat heart. Gates with positive events were drawn based on the unstained samples. Filled histograms represent test samples, and unfilled ones show the fluorescence signal for unstained controls.

CD34, PDGFR-α and PDGFR-β expression levels in the whole cell population are shown in Figure7 as percentage positive of the whole cell population. The PDGFR-α^+^ population varied from 80% to 91% of the total number of cells, with an average value of 87%. The CD34^+^/PDGFR-α^+^ population varied from 29% to 41%, while CD34^+^/PDGFR-β^+^ represented 28–36% (data not shown). The entire cell population shows a high expression level for PDGFR-α (‘bright’ events) and a lower expression level for CD34 and PDGFR-β (‘dim’ events; Fig.8). CD34/PDGFR-α positive events accounted for 30.25% of the cell population (data not shown).

## Discussion

For the present time, TEM remains the only method to precisely identify TCs 25. Recently, using advanced focused ion beam scanning electron microscopy, the ultrastructural anatomy and 3D reconstruction of human cardiac TCs have been reported 42. However, the immunocytochemical phenotypes of TCs are also critical markers for TCs. Generally, double immunofluorescent staining for CD34/PDGFR-β, CD34/Vimentin or CD34/c-Kit are considered to be the markers for TCs although CD34/c-Kit is currently at debate 25,43. In addition, double immunofluorescent staining for CD34 and PDGFR-α is considered to be a specific immunohistochemical marker for TCs in gastrointestinal tract 7.

However, it has been recently proposed that CD34/PDGFR-α was actually a specific marker for TCs including cardiac TCs although the direct evidence is still lacking 25. Here, we showed that cardiac TCs were double positive immunostaining for CD34/PDGFR-α in primary culture and CD34/PDGFR-α positive cells (putative cardiac TCs) existed in mice ventricle and human cardiac valves including mitral valve, tricuspid valve and aortic valve. Telocyte-enriched rat cardiac interstitial cell population showed a high expression level for PDGFR-α and 30.25% of the entire population was positive for CD34/PDGFR-α. Overall, this study reported that cardiac TCs were double positive immunostaining for CD34/PDGFR-α for the first time.

Cardiac TCs have been reported to be able to regulate cardiac stem/progenitor cells, angiogenesis, and anti-fibrosis by heterocellular junctions and secreting extracellular vesicles containing microRNAs 24,25,44–51. A deeper understanding of the immunocytochemical phenotypes of cardiac TCs might help better develop cardiac TCs as a novel source for cardiac repair as preliminarily demonstrated in cardiac infarction 52–55.
